# Temperature-sensitive heparin-modified poloxamer hydrogel with affinity to KGF facilitate the morphologic and functional recovery of the injured rat uterus

**DOI:** 10.1080/10717544.2017.1333173

**Published:** 2017-06-02

**Authors:** He-Lin Xu, Jie Xu, Si-Si Zhang, Qun-Yan Zhu, Bing-Hui Jin, De-Li ZhuGe, Bi-Xin Shen, Xue-Qing Wu, Jian Xiao, Ying-Zheng Zhao

**Affiliations:** a Department of Pharmaceutics, School of Pharmaceutical Sciences, Wenzhou Medical University, Wenzhou City, PR China;; b First Affiliated Hospital, Wenzhou Medical University, Wenzhou City, PR China

**Keywords:** Keratinocyte growth factor, intrauterine adhesion, temperature-sensitive hydrogel, uterus injury, sustained-release

## Abstract

Endometrial injury usually results in intrauterine adhesion (IUA), which is an important cause of infertility and recurrent miscarriage in reproductive women. There is still lack of an effective therapeutic strategy to prevent occurrence of IUA. Keratinocyte growth factor (KGF) is a potent repair factor for epithelial tissues. Here, a temperature-sensitive heparin-modified poloxamer (HP) hydrogel with affinity to KGF (KGF-HP) was used as a support matrix to prevent IUA and deliver KGF. The rheology of KGF-HP hydrogel was carefully characterized. The cold KGF-HP solution was rapidly transited to hydrogel with suitable storage modulus (G′) and loss modulus (G″) for the applications of uterus cavity at temperature of 33 °C. *In vitro* release demonstrated that KGF was released from HP hydrogels in sustained release manner for a long time. *In vivo* bioluminescence imaging showed that KGF-HP hydrogel was able to prolong the retention of the encapsulated KGF in injured uterus of rat model. Moreover, the morphology and function of the injured uterus were significantly recovered after administration of KGF-HP hydrogel, which were evaluated by two-dimensional ultrasound imaging and receptive fertility. Not only proliferation of endometrial glandular epithelial cells and luminal epithelial cells but also angiogenesis of injured uterus were observed by Ki67 and CD31 staining after 7 d of treatment with KGF-HP hydrogel. Finally, a close relatively relationship between autophagy and proliferation of endometrial epithelial cells (EEC) and angiogenesis was firstly confirmed by detecting expression of LC3-II and P62 after KGF treatment. Overall, KGF-HP may be used as a promising candidate for IUA treatment.

## 1. Introduction

The endometrium is a mucosal tissue comprised of epithelial, stromal, and vascular components that undergo hormonally driven changes in structure and function during the normal menstrual cycle. Healthy uterus endometrium is essential for embryo implantation and the maintenance of pregnancy (Fernandez et al., 2006). However, when healthy uterus suffered from trauma, curettage, or gynecological disease, such as myomectomy, endometritis, endometrial tuberculos, intrauterine adhesion (IUA) was liable to be acquired, resulting in miscarriage, or infertility (Muller et al., 2011; Radivojsa et al., 2013; Ding et al., 2014). IUA is the most common endometrial syndrome secondary to endometrial injury. Current strategies focus on early intervention to reduce scar formation, promote the regeneration of damaged intima, and maintain a special three-dimensional structure of the uterus (Zhao et al., 2016). At present, placement of intrauterine device (IUD) and application of artificial hormone therapy with high doses of estrogen are used in combination to prevent occurrence of IUA and promote endometrial restoration and regeneration in clinic (Radivojsa et al., 2013). Although this therapeutic mode may archive some primary symptomatic relief, the recurrences and even some severe side effects, such as re-adhesion is often not avoided. For example, after treatment with this strategy, the recurrence rate was still up to 62.5% in severe IUA (Tian et al., 2010), and the pooled pregnancy rate in women conceiving naturally or with assisted reproduction technology is only 42.8–57.0% (Tian et al., 2010; Zhao et al., 2016; Wang et al., 2017).

Recently, as an alternative, hydrogels with three-dimmensional architecture was more advantageous to prevent adhesion formation compared with IUD, because of its good affinity and compatibility to the endometrium. Additionally, it was easily also administrated to uterine cavity through perfusion with a minimally invasive injury (Meyvis et al., 2001; Yamamoto et al., 2001 ). For instance, a hydrogel of polyvinyl alcohol and carboxymethyl cellulose (PVA/CMC) was found be effective to prevent postoperative IUA formation in a rabbit uterus model (Muller et al., 2011). Besides, some therapeutic factors, such as stem cells, trophic factors can be incorporated, or absorbed to the scaffold of hydrogel for sustained release. Bone marrow derived mesenchymal stem cells (BM-MSCs) were loaded onto degradable collagen hydrogel for the injured rat uterus (Ding et al., 2014). At four weeks after the transplantation, collagen/BM-MSCs hydrogel increased proliferative abilities of uterus endometrial and muscular cells, facilitated microvasculature regeneration, and restored the ability of endometrium to receive the embryo and support its development to a viable stage. However, immune rejection of transplanted cells remains an important issue to overcome for therapeutic efficacy.

In contrast, angiogenesis-enhancing cytokines and growth factors secreted by stem cell have received increasing attention. Keratinocyte growth factor (KGF), also known as fibroblastic growth factor-7 (FGF-7), is a stromally derived, secreted peptide that acts as a mitogen for epithelial cells in culture (Chomiski et al., 2016; Poorebrahim et al., 2017). In previous studies, KGF has been proved to facilitate regeneration and healing of wound in injured skin, oral mucosa, and cornea (Eming et al., 1999; Finch & Rubin, 2004). Endometrial KGF levels were increased by progesterone treatment in the macaque and mouse and are elevated during the luteal phase of the menstrual cycle in women (Slayden et al., 2000). It was discovered that exogenous KGF stimulate spiral artery growth and inhibit glandular apoptosis during luteal-follicular transition, but did not affect cell proliferation in the endometrium or block menstrual sloughing and bleeding in normal uterus. The signal mechanism of KGF in regulating epithelial cell homeostasis has been poorly understood. Furthermore, it was not clear whether exogenous KGF can facilitate the morphologic and functional recovery of the injured uterus.

Poloxamers are nonionic, poly (ethylene oxide)–poly (propyleneoxide)–poly (ethylene oxide)copolymers (PEO–PPO–PEO), which form micellar solution at low concentrations and clear thermo reversible hydrogels at high concentrations (Xu et al., 2016). They have been widely used in various drug delivery systems due to their advantages, such as low toxicity and biocompatibility (Yamamoto et al., 2001; Zhang et al., 2015). Heparin can stabilize various growth factors or cytokines through a specific receptor binding, and thus control their release behavior (Niu et al., 2008; Tian et al., 2010). In our previous study, a new heparin–poloxamer conjugate (HP) was synthesized and its physicochemical properties were investigated (Tian et al., 2010). HP still exhibited a good gelation profile and its solution-gel transition temperature was not compromised compared with poloxamers (Wu et al., 2016). Importantly, HP still exhibited a strong affinity to heparin-binding growth factor. Our previous studies have exploited HP hydrogel to incorporate various growth factors, such as nerve growth factor (NGF) (Zhao et al., 2016), acidic fibroblast growth factor (aFGF) (Wang et al., 2017), and basic fibroblast growth factor (bFGF) (Xu et al., 2016) for prolonging its half-lives *in situ*. KGF, a heparin-affinitive growth factor, are also confronting with tremendous challenges in clinic because of its short half-life, poor stability, and low permeability (Belleudi et al., 2011).

In this study, temperature-sensitive HP was also used as a hydrogel-forming material to support injured uterus and delivery of KGF. It was expected that *in situ* gelation of HP solution after exposure to body temperature can make encapsulated KGF adhere to injured zone, avoiding rapid discharge with the secreted physiological mucus, and then be released in a sustained release manner. The rheological and mechanism properties of temperature-sensitive HP hydrogels were firstly investigated, which were important parameters to support injured lumen of uterus. Afterward, the sustained-release of encapsulated KGF from HP was also evaluated *in vitro* and *in vivo*. And then, the therapeutic effect of KGF-HP hydrogel on the morphologic and functional recovery of the injured uterus was further evaluated in rat model with partial full thickness injury of uterus. Finally, the underlining molecular mechanism was also probed.

## 2. Material and methods

### 2.1 Materials

KGF was provided by (Anhui Xinhuakun Co, Tongling City, China). Heparin–poloxamer conjugate (HP) was synthesized by our laboratory according to method as previously reported (Zhao et al., 2013). Dulbecco’s Modified Eagle’s Medium/nutrient mixture F-12 ham’s (DMEM/F12) and fetal bovine serum (FBS) were purchased from Invitrogen (Carlsbad, CA). CCK-8kit was purchased from Dojindo (Minato-ku, Japan). Fluorescein isothiocyanate (FITC) was ordered from Sigma (Sigma Aldrich, St. Louis, MO).Antibodies of Ki67, TGF-β, and CD31were purchased from Abcam (Abcam, CB, United Kingdom). Anti-p62 and microtubule-associated protein-1 light-chain 3 (LC3) were purchased from Cell Signaling Technologies (Danvers, MA).

### 2.2 Preparation of KGF-HP temperature-sensitive hydrogels

KGF-HP hydrogels were prepared by a sol-gel transition method as reported in previous publication. Briefly, the lyophilized powder of HP was firstly dissolved in cold PBS solution at a concentration of 17% (w/w), and then the concentrated KGF stock solution (1 mg/ml of KGF in pH7.6 PBS) was added to cold HP solution and mixed at 4 °C under gentle stirring. The KGF-HP solution becomes hydrogel in thermostat bath at 37 °C within minutes of incubation.

### 2.3 Sol-gel transition temperature and rheology of KGF-HP hydrogels

Sol-gel transition temperature of KGF-HP hydrogel was reflected by detecting viscosity of KGF-HP solution using a coaxial cylinder rheometer (NDJ-8 S, Shanghai Pingxuan Co, Shanghai, China). The apparent viscosity was measured at different temperature from 25 to 45 °C, and the curves of apparent viscosity were plotted against temperature. Sol-gel transition temperature was indicated by the viscosity curve at maximum slope.

Rheological measurements of KGF-HP hydrogels were carried out using the hybrid rheometer (Discovery HR-2, TA Corporation, America) in the temperature range of 10–45 °C. The program settings of elastic modulus and viscous shear modulus are as follows: the flat plates with diameter of 12 mm, shear frequency at 10 rad/s and 1% of shear strain.

### 2.4 Scanning electronic microscopy of KGF-HP hydrogel

The surface morphology of the KGF-HP temperature-sensitive hydrogels was observed by scanning electron microscope (SEM). A mixture of hydrogels were frozen and lyophilized with a vacuum freeze-dryer for 24 h. The lyophilized specimens were sputter-coated with gold, and the surface was then observed by scanning electron microscope (H-7500, Hitachi, Japan).

### 2.5 *In vitro* KGF release from hydrogel

*In vitro* release of KGF was investigated by the dynamic dialysis method depicted in literature (Choi et al., 2016). Briefly, 1 ml of cold KGF-HP solution (equivalence of 2.5 mg/ml KGF) or free KGF solution (2.5 mg/ml KGF) was placed in the dialysis bag (MW, 100 kDa) and dialyzed against 20 ml of pH7.4 PBS containing 0.1% w/v BSA. The whole set was placed on a magnetic stirrer at 37 °C. The cold KGF-HP solution in dialysis bag was rapidly changed to be hydrogel status within 3 min. At each predetermined time intervals, 0.1 ml of aliquot in release medium was withdrawn for ELISA analysis and replaced by an equal volume of fresh medium to maintain a constant volume. The cumulative release percentage (%) was determined by dividing the cumulative amount of KGF recovered in the release medium at each time point by the total amount of KGF in 1 ml of KGF-HP hydrogel.

### 2.6 *In vitro* cellular proliferation

#### 2.6.1 Extract and separation of primary endometrial epithelial cells

The endometrial epithelial cells (EEC) were extracted and separated from BALB/C female mice. Mouse was sacrificed by 10% chloralic hydras (3.5 ml/kg) and the uterus was collected. The tissue was cut into small pieces, and then washed with D-hanks for three times, digested indigestive liquid containing 0.1% collagenase, 0.25% trypsin-EDTA, and 0.1% hyaluronic acid enzyme for 1 h under gentle-shaking at 37 °C. Afterward, DMEM/F12 was added to terminate the digestive reaction and the digestive tissue was filtered through cell strainers (size of 150 μm, BD Bioscience) to remove the residual tissue crumb. EEC cells were isolated by centrifugation at 500 rpm for 5 min and washed with serum-free DMEM/F12 medium for three times. Finally, the isolated EEC cells were re-suspended in DMEM/F12containing 10% fetal bovine serum, 1% antibiotics (100 U/ml penicillin, 100 ng/ml streptomycin), and cultured for 24 h.

#### 2.6.2 Promoting proliferation of H_2_O_2_-induced injured EECs by KGF

The cultured EEC cells were damaged by incubating with DMEM/F12 medium supplemented with 1700 μmol/L H_2_O_2_ for 4 h, as depicted in previous publication (Zhao et al., 2016). The effect of KGF concentration on proliferation of injured EEC cells was firstly screened. The injured EEC cells were incubated with DMEM/F12 medium containing various dose of KGF (5, 10, 20, 50, 80, 100, and 200 ng/ml) for 2, 4, and 8 h. After incubation, the culture medium was removed and Cell Counting kit-8 (CCK-8, Dojindo Laboratories Inc., Japan) was added for further incubation of 2 h. Afterward, the absorbance was measured at 450 nm by a microplate reader. Second, the proliferation effect of various KGF formulations on injured EEC was also tested at KGF concentration of 80 ng/ml. As control, the effect of vehicle (PBS), HP hydrogel alone, and estradiol (E_2_, 80 ng/ml) on the proliferation of injured EEC cells were also evaluated.

#### 2.6.3 The cellular uptake of KGF by H_2_O_2_-induced injured EECs

Cellular uptake of various KGF formulations by H_2_O_2_-induced injured EECs was probed to determine whether availability of KGF, either free form or HP-encapsulated form, to EEC cells. To track destiny of KGF, fluorescent FITC was covalently tagged to amino terminal of KGF through the reported method in literature (Xu et al., 2016). H_2_O_2_-induced injured EECs were grown on coverslips in 6-well plates at a density of 7 × 10^3^ cells/well for 24 h. The culture medium was removed and replaced with 200 μl of DMEM/F12 medium containing FITC-labeled KGF (FITC-KGF, 80 ng/ml of equivalent KGF) or FITC-KGF-HP hydrogel (FITC-KGF, 80 ng/ml of equivalent KGF). After 1, 2, and 4 h of incubation, the culture medium was removed, and EEC cells were rinsed with PBS for three times, fixed with 4% paraformaldehyde and stained with DAPI for observation by confocal laser microscopy (A1 PLUS, Japan). For quantitative analysis, EEC cells were collected by trypsin digestion, washed with PBS for three times, and suspended in 0.5 ml of PBS for analysis by flow cytometry (FACSCalibur FCM, Becton Dickinson, San Jose, CA).

### 2.7 *In situ* retention of KGF-HP hydrogel

To investigate in situ retention of various KGF formulations in uterus lumen, 30 μl of FITC-KGF solution or cold FITC-KGF-HP solution (equivalence of 2.5 mg/ml KGF) were injected into intrauterine cavity of healthy rats. At 2, 8 h, 1, 3, and 7d after administration, the rats were sacrificed, and the uterus was collected, followed by washing surface of collected uterus with saline for three times. Afterward, fluorescence images of intact uterus were collected using an in vivo bioluminescence imaging. For imaging the uterus treated with FITC-KGF-HP hydrogel, all procedures were handled under thermostat environment with 37 °C.

### 2.8. Repair of the injured intrauterine

#### 2.8.1 Animal model with intrauterine injury

Female Sprague-Dawley rats (220–250 g) were purchased from the Animal Center of Chinese Academy of Sciences (Shanghai, China). Experiments were performed in accordance with Guide for the Care and Use of Laboratory Animals from National Institutes of Health and approved by the Animal Care and Use Committee of Wenzhou Medical University. Rats were maintained on SPF level of environment.

Vaginal smears were obtained daily between 03:00 and 04:00 pm. Female Sprague-Dawley rats with four consecutive 4-d estrus cycles were screened to establish the animal model with IUA according to the method depicted in publications (Liu et al., 2013). Rats were anesthetized by an intra-peritoneal injection of 10% chloralic hydras (3.5 ml/kg), and excised in a low midline abdomen, exposing the uterus. The uterus were operated on through a 0.5-cm transverse incision in the lowest one-third of the connection between the middle and distal uterus and then the endometrial lining of 1-cm uterus on supine right uterus was scraped using a 2-mm endometrial curette until the uterus wall became rough and with obvious endometrial swelling. Afterward, the rectus fascia and skin were then repaired with sutures. The surgical procedures were performed under sterile conditions. For the control group, only abdomen was exposed by making an incision, without scalping the uterus. IUA group animals received the same surgical procedures and were given saline.

#### 2.8.2 Local delivery of KGF-HP to the injured intrauterine of rats

To investigate in the *vivo* functional and morphologic recovery of the injured intrauterine, rats with uterus injury were randomly divided into the following groups, including sham group, IUA group, group treated with HP hydrogel, group treated with E_2_ (2.5 mg/ml, Estradiol benzoate), group treated with KGF (2.5 mg/ml), group treated with KGF-HP hydrogel (equivalence of 2.5 mg/ml). A total of 30 μl of various formulations were administered in situ via cavity injection. All rats received intramuscular injections of penicillin twice a day for 3 d after the surgery. At 3 or 7 d after treatment, the rats were sacrificed and the hearts were perfused with saline followed by 4% paraformaldehyde, and then the uterus was collected and frozen in −80 °C refrigerator.

#### 2.8.3 Two-dimensional ultrasound imaging

Usually, the two-dimensional ultrasound technology was used to observe the health status of female endometrium in clinical diagnosis. At 7 d after treatment with various formulations, rats were anesthetized by 10% chloralic hydras (3.5 ml/kg, peritoneal injection), their abdominal hairs were removed and their uterus were imaged by the Vevo2100 (Visualsonics Inc., Toronto, Canada) and a 21-MHz (MS250) probe at a mechanical index of 0.43.

#### 2.8.4 Morphologic recovery of the injured uterus

**Histological analysis:** After treatment with various formulations, the injured uterus were collected, fixed with 10% chloralic hydras, and dehydrated in graded alcohols, and embedded in paraffin. The embedded tissues were consecutively sliced into 5-mm thick sections, which were routinely stained with hematoxylin and eosin (HE staining). The number of glands on each HE-staining section was counted in four random fields, and the mean number was calculated. The thickness and the morphology of endometrium were examined and measured under a light microscope.

Tissue sections were also immunolabeled with Masson’s trichrome. The degree of endometrial fibrosis was quantified by measuring the area ratio between endometrial stromal fibrosis and the endometrial area using a quantitative image analysis system (Image-Pro Plus software; Media Cybernetics, Bethesda, MD) under a magnification of 200.

**Transmission electron microscope (TEM) analysis:** Transmission electron microscopy was performed to assess microstructural changes in the injured uterus after various treatments. The uterus from rats with sham, IUA, HP alone, KGF, KGF-HP groups was cut into pieces of 1 × 1 mm, and perfused with normal saline followed by a fixture solution containing 2.5% glutaraldehyde. The uterus tissues were fixed with 2.5% glutaraldehyde at 4 °C for 2 h and washed three times for 30 min in 0.1 M PBS. Afterward, the samples were post-fixed with 1% OsO_4_ for 2 h and dehydrated in an ascending gradual series (50–100%) of ethanol and infiltrated with propylene oxide. Specimens were embedded in Araldite for cross sections. Ultra-thin sections were cut by Leica Ultra cut UCT Ultramicrotome. The sections were double-stained with uranyl acetate and lead citrate for contrast staining. All sections were observed under transmission electron microscopy (JEM 1400).

#### 2.8.5 Functional recovery of the injured uterus

The function of the regenerative uterus was assessed by testing whether it was receptive to a fertilized ovum and was able to support embryo development to the late stages of pregnancy. Two weeks post-procedure, rats (*n* = 10 uterus of sham group, and *n* = 18 uterus of other groups) were mated with male Sprague-Dawley rats. The day of vaginal plug presence was considered as gestation day 0. Rats were euthanasia at gestation day 18, and uterus was examined for the presence of embryos.

### 2.9 Staining of Ki67 and CD31

Immunohistochemistry staining was performed on deparaffinized sections. Primary anti-Ki67 (ab16667, 1:200, Abcam) and anti-CD31 (ab28364, 1:200, Abcam) antibodies were diluted with PBS containing 1% BSA. After dewaxed and hydrated, the nonspecific antigens of the specimens were blocked with 3% H_2_O_2_ for 15 min and with 5% bovine serum albumin (BSA) (Beyotime) in PBS for 45 min at 37 °C, respectively. Subsequently, primary antibodies were incubated at 4 °C overnight followed by secondary antibodies for 2 h at 37 °C. And then the reaction was stopped with DAB chromogen kit (ZSGB-BIO, Beijing, China) followed by hematoxylin staining. Images were acquired using Nikon positive position microscope (Nikon, 80i, Tokyo, Japan). The numbers of positive-staining cells were counted and quantified by optical density using Image-Pro plus (Reindeer Graphics, Inc., Asheville, NC). Capillary vessels were also counted from at least three randomly selected fields each section under a magnification of 400.

Immunofluorescence staining of CD31 was also performed on deparaffinized sections. Sections were stained with anti-CD31 followed by goat anti-rabbit IgGAlexa Fluor^®^ 647 (ab150083, 1:1500, Abcam, Cambridge, United Kingdom). Nuclei were stained using DAPI (Beyotime). Angiogenesis was observed by confocal laser microscopy (A1 PLUS, Japan). The number of CD31-positive cells in the tissue was counted by optical density using Image-Pro plus.

### 2.10 West blotting analysis

Uterus segment (0.5 cm length) at the lesion was dissected and soon stored at −80 °C for western blotting. Briefly, the tissue was homogenized in modified RIPA buffer containing protease inhibitor cocktail (10 mg/μl, GE Healthcare Biosciences, PA).The complex was then centrifuged at 12,000 rpm for 15 min and the supernatant was collected for assay. After quantification by BCA assay, 50 μg of proteins were separated by sodium dodecyl sulfate (SDS)-polyacrylamide gel and transferred to PVDF membrane (Bio-Rad, Hercules, CA). The blots were probed with the indicated primary antibodies, LC-3(1:500; Cell Signaling Technology; Danvers, MA), and anti-P62(1:1000; Cell Signaling Technology; Danvers, MA).Next, the membranes were incubated with a goat-anti-rabbit secondary antibody for 2 h at room temperature, and bands detected using the enhanced chemiluminescence (ECL) kit (PerkinElmer, Waltham, MA). Band intensity was quantified using the Image Lab 3.0 software (Bio-Rad, Hercules, CA).

### 2.11 Statistical analysis

Two independent observers performed the histological measurement with the average was taken for subsequent analysis. Data were presented as mean standard deviation (SD). Multiple comparisons were determined by one-way analysis of variance using Graph Pad Prism version 5.01 for Windows (GraphPad Software, San Diego, CA). Student’s t-tests were performed to determine the significance of differences between pairs. One-way analysis of variance (ANOVA) was utilized to determine significant differences between multiple groups. *χ*^2^ test were performed for comparison of pregnancy rate. *p* < .05 was considered statistically significant (*p < .05 or **p < .01).

## 3. Results

### 3.1 Characterization of HP hydrogel

**Figure 1.**
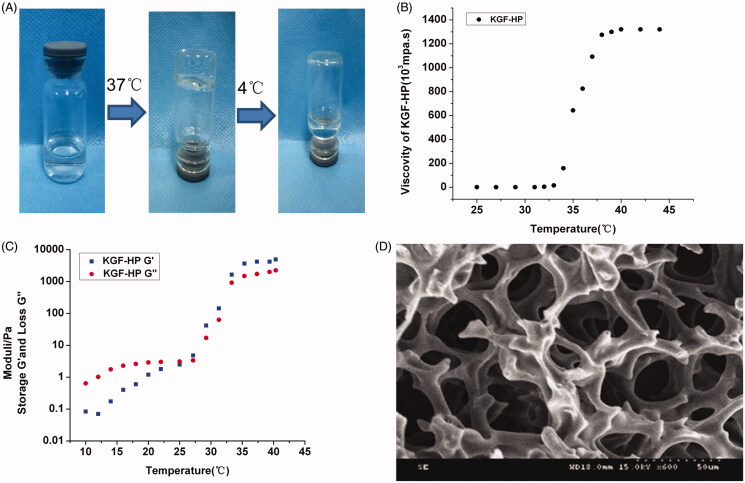
*In vitro* characteristics of KGF-HP hydrogels with KGF concentration of 2.5 mg/ml, (A) Temperature-dependent appearance, (B) the curves of apparent viscosity against temperature, (C) Storage (G') and loss (G'') moduli of KGF-HP as a function of temperature and (D) SEM images of the lyophilized KGF-HP hydrogel.

HP hydrogel were prepared by a sol–gel transition method. Gelation temperature and viscoelastic property of HP hydrogel were investigated after incorporation of KGF. As shown in Figure 1(A,B), even KGF concentration in HP solution was up to 2.5 mg/ml, gelation of KGF-HP solution was not compromised and its gelation temperature was still ca. 33 °C, at which HP material was gelled. The apparent viscosity of KGF-HP hydrogel was increased at first and then reached a plateau value as temperature increased. As temperature increased from 35 to 37 °C, the apparent viscosity of KGF-HP hydrogel increased from 8000 to 10,000 mPa s, maintaining a stable viscosity of 12,000 mPa s at the plateau (Figure 1(B)).The apparent viscosity of the hydrogel suitable for *in situ* application was considered to be approximately 10,000 mPa s in the literature (Raposo et al., 2013). Therefore, KGF-HP hydrogel was suitable for in situ intrauterine application. Viscoelastic materials can be characterized by dynamic experiments when a sinusoidally oscillating stress or strain is applied to the material. The storage modulus (G′) and loss modulus (G″) parameters are used, where G' reflects the elastic response and G'' viscous response, respectively. Results of rheology tests shown in Figure 1(C), it indicated that the viscoelastic behavior of KGF-HP hydrogel changed as a function of temperatures. As temperature below 30 °C, the KGF-HP system has a more viscous behavior and could be easily perfused into intrauterine lumen, while its elasticity was more significant as temperature above 33 °C, making secure coverage and retention of KGF-HP hydrogel the injured uterus *in vivo*. The morphology of KGF-HP hydrogels was also observed under SEM. KGF-HP hydrogel displayed a porous structure resembling a cribriform plate (Figure 1(D)), which was favorable for the localized and sustained delivery of KGF.

### 3.2 *In vitro* release and *in situ* retention of KGF from KGF-HP hydrogel

**Figure 2.**
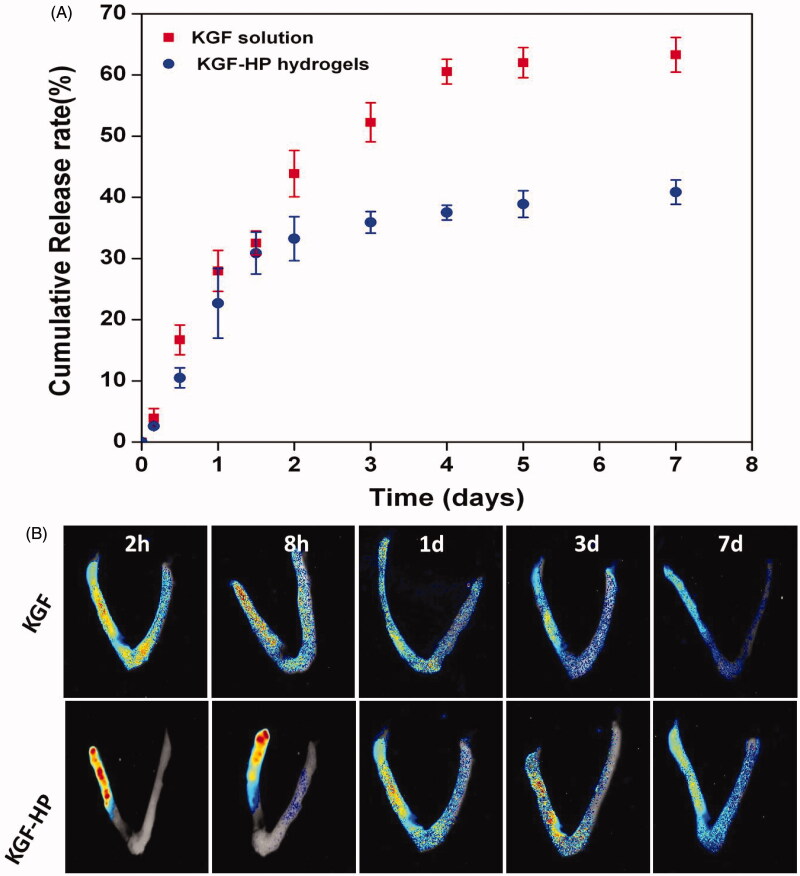
(A) The cumulative release profile of free KGF solution and KGF-HP hydrogel; (B) Representative fluorescence images of intact uterus from rats after treatment with FITC-labeled KGF or KGF-HP hydrogel.

*In vitro* release of KGF from KGF-HP hydrogels was examined. Results displayed in Figure 2(A), a sustained release profile was exhibited for KGF-HP hydrogel especially at late stage, while free KGF was rapidly released by diffusing out the dialysis membrane. KGF release was quantified for up to 7 d for both free KGF solution and KGF-HP hydrogels. A burst release of free KGF solution about 28% was observed on day 1, and another 37% was released over the following 6 d. The cumulative release of free KGF solution was high up to 65% and about 35% of KGF may either remain in dialysis bag without release or be partly degraded during release process. For KGF-HP hydrogel, despite approximately 22% of KGF was released from KGF-HP hydrogel within initial 1 d, a sustained release profile was displayed at late stage. Approximately 35% of KGF was released within 3 d, and only 38% of KGF was released even at 7 d. The rapid release for KGF-HP hydrogel at initial stage was due to part of KGF resided inside near surface of HP hydrogel. In contrast, the very slow release of KGF at late stage was contributed to the porous network of HP hydrogel and the strong binding between heparin and KGF. The phenomenon that these factors resulted in a sustained-release profile of NGF and aFGF has also been reported in these publications (Wang et al., 2017).

Rat uterus is Y-type pipeline structure and connected on both sides of the uterus cavity. The exogenous substance inside uterus was easily moved and cleared by uterus mucus. KGF was successfully labeled by fluorescent FITC without compromise of KGF structure according to the method depicted in previous study. FITC-labeled KGF solution or KGF-HP hydrogel was administrated to the injured uterus lumen of rat, and its retention and distribution was also investigated by *ex vivo* fluorescence imaging. The rats were administrated with FITC-KGF solution or FITC-KGF-HP hydrogels, and the fluorescence images of intact uterus were taken at different time points. Results are shown in Figure 2(B). Strong fluorescence was retained at the injured zone (right horn of uterus) post-administration of KGF-HP hydrogel for a long time. Even at 8 h post-administration, the fluorescence intensity was localized in the injured area without significant decrease. However, upon administration of KGF solution, it rapidly diffused along lumen of injured uterus to left horn of uterus within 8 h and residual KGF localized in injured zone decreased with time. As the time increased to 1 and 3 d, the fluorescence at the uterus decreased for both of free KGF solution and KGF-HP hydrogels, but KGF-HP hydrogel group exhibited a relatively higher fluorescence in damaged zone than free KGF solution. Even at 7 d, there was still a strong residual fluorescence in the uterine cavity for KGF-HP hydrogels, but a faint fluorescent was observed in KGF solution group. These indicated that HP hydrogel significantly prolonged the retention of KGF in uterus lumen. The long retention of KGF was very helpful to facilitate the recovery of the injured rat uterus.

### 3.3 Promoting proliferation of theH_2_O_2_-induced injured EECs by KGF

To further confirm the effects of KGF on protecting the injured EECs, we applied H_2_O_2_ to the EECs and measured the proliferation of the injured EECs after treatment with KGF formulations. In preliminary experiment, it was suggested that exposure of EECs to 1700 μM of H_2_O_2_ for 4 h produced cell death of half of the cultured EECs, producing significant injury of cells. After H_2_O_2_-induced injury, the EECs was immediately treated with various concentration of KGF for various time, the proliferation of EECs was measured by CCK-8 kit. The results are shown in Figure S1(A). Compared with vehicle (PBS), the dose-dependent and time-dependent proliferation of injured EECs was exhibited. Interestingly, an optimal concentration of KGF to promote cell proliferation was identically exhibited to be 80 ng/ml whatever time of incubation. The protective effect of KGF-HP hydrogel (equivalence of 80 ng/ml KGF) was also detected and results is shown in Figure S1(B). The HP alone did not exhibit any activity on cell proliferation as PBS, which was similar to previous results (Zhao et al., 2016). Interestingly, KGF either free form or HP hydrogel-encapsulating, exhibited an enhanced profanation activity on injured EECs than E_2_, a typical agent promoting cell proliferation. Moreover, the proliferation activity of KGF-HP was significantly higher than those of free KGF solution after 24 or 48 h of incubation, but not 12 h. This may be due to the fact that the half-lives of KGF encapsulated in HP was pronged when its sustained release of KGF from hydrogels, which was very beneficial to clinical therapy with single dose.

### 3.4 Cellular uptake

To investigate cellular uptake of KGF by the injured EEC, the fluorescence distribution of KGF in EEC cells after 1, 2, and 4 h of incubation is exhibited in Figure S2(A). After 1 h of incubation, free KGF solution rapidly distributed in the bulk cytoplasm and exhibited a strong fluorescence. As time of incubation increased, the fluorescent signal was increasing. The strongest fluorescence was observed at 2 h followed by a decreased fluoresce at 4 h. These suggested that free KGF was fast uptake by EECs, and then rapidly cleared from cytoplasm. In contrast, only a few KGF were distributed inside cytoplasm in the case of KGF-HP hydrogels, exhibiting a week fluorescent signal after 1 or 2 h of incubation. As time of incubation increased, the fluorescent signal was continuously increasing. After 4 h of incubation, EEC cells treated with KGF-HP hydrogel showed the highest KGF fluorescence inside cytoplasm. These results indicated that the continuous accumulation of KGF in EECs after incubated with KGF-HP hydrogels. The higher KGF retention by HP hydrogel was contributed to the sustained release of KGF from KGF-HP. The release rate of KGF from hydrogel may reach a balance with that of KGF clearance from cytoplasm, which helped maintain a high-KGF concentration inside cytoplasm for a long time. The retained KGF in cytoplasm for 4 h was also quantified using flow cytometry. The similar results are obtained as shown in Figure S2(B).The amount of KGF inside EEC cells treated with KGF-HP hydrogel for 4 h was approximately 3-fold higher than that of cells treated with free KGF solution (Figure S2(C)). These further confirmed that the sustained-release of KGF from HP hydrogel was beneficial to maintain a higher KGF concentration inside cytoplasm because of preventing its rapid clearance.

### 3.5 Recovery of the injured endometrial morphology

#### 3.5.1 Two-dimensional ultrasound imaging

**Figure 3.**
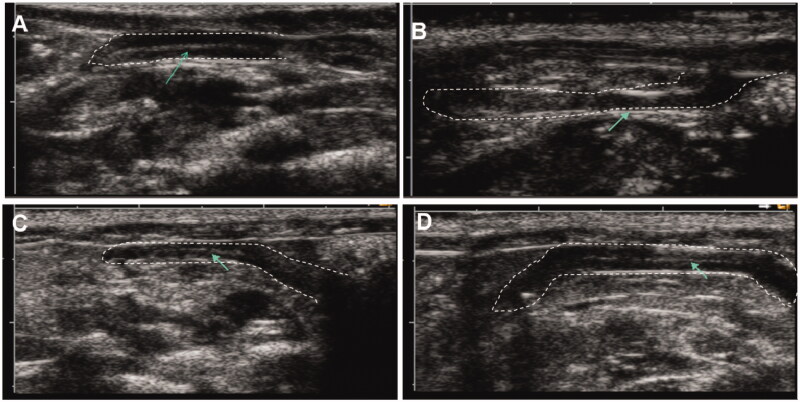
Representative Two-dimensional ultrasound images of rat uterus, (A) sham group, (B) vehicle-treated group (IUAgroup), (C) KGF solution-treated group, and (D) KGF-HP hydrogel-treated group.

The whole endometrium structure was observed by 2D ultrasound imaging. The images are shown in Figure 3. The uterus of sham group exhibited a continuous echogenic signal, which reflected a bright white endometrial line centered in the uterus cavity. After injury of the uterus, echogenic signal of endometrium structure was not detected near uterus cavity and endometrial line was not observed by ultrasound imaging, indicating appearance of IUA. In contrast, at 7 d after treatment with either KGF solution or KFG-HP hydrogel, echogenic signal of endometrium structure was recovered and a bright white endometrial line was observed, suggesting effective recovery of the injured endometrial morphology.

#### 3.5.2 Histological evaluation of injured uterus

Histological morphology of injured uterus was also evaluated by HE and Masson staining. Results are shown in Figure S3. Uterus of sham group exhibited a few glands in the submucosa and basal layer, and little deposition of collagen fiber at both time points, indicating its normal endometrial morphology. However, comparing with sham group, few glands were observed and obvious collagen fibers were deposited in injured uterus of IUA group, indicating poor morphologic recovery of injured uterus even after 3 or 7 d. There was not obvious improvement in uterus in glands and deposition of collagen fiber from injured uterus treated with HP alone after 3 or 7 d. By contrast, after 3 d for rats treated with free KGF solution or KGF-HP hydrogel, a few glands were restored and gland number was significantly higher than that of IUA group (Figure S3(A,B)). Moreover, especially to deserve to be mentioned, repair of glands in these groups was significantly better than that in E2 group. Besides, deposition of collagen fiber for KGF group or KGF-HP hydrogel was of little and fibrosis area was significantly reduced compared with IUA group or HP group (Figure S3(C)), indicating the effective recovery of histologic morphology. It was also reported that reduction of collagen fiber, an important factor to determine the repair effect, was exhibited after recovery of injured uterus in previous publications [59 − 61]. Over time, at day 7, there exhibited a more significant increase in the number of glands and a decrease in fibrosis area compared with results at 3 d for treatment with KGF-HP group, KGF group, and E_2_ group (Figure S3(D)). Despites of these, either improvement of glands or inhibition of collagen fiber after treatment with KGF-HP hydrogel was significantly better than that treated with free KGF at 7 d (Figure S3(E,F)). This was due to the fact that KGF-HP hydrogel can deliver an appropriate amount of KGF to injured zone in a sustained release profile as biomimetic native modes of wound healing.

#### 3.5.3 Transmission electron microscope (TEM) of injured uterus

**Figure 4.**
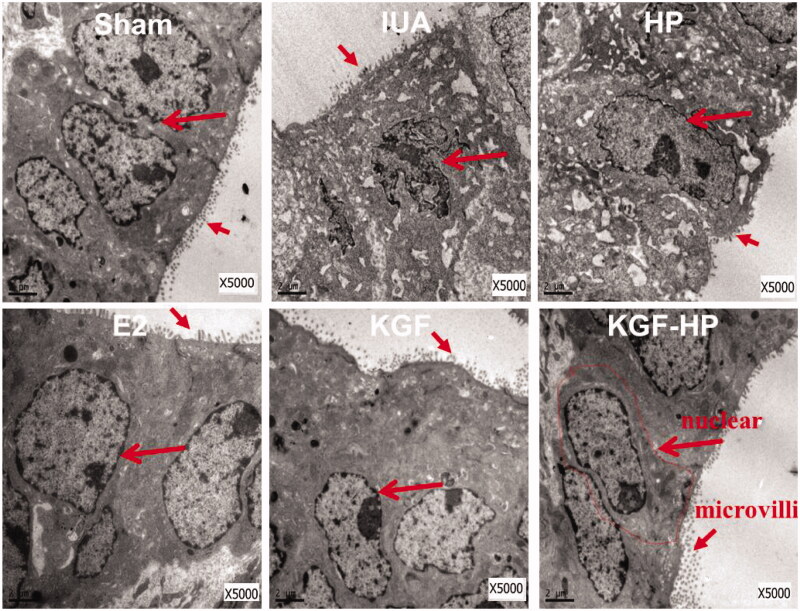
Transmission electron microscopy (TEM) images show the microstructure of the endometrial epithelial cells. The structure was monolayer columnar epithelial cell; Microvilli on the surface epithelium; Scar= 2 μm. (**p < .05, **p < .01; *n* = 3).

The ultrastructure of endometrium was also observed by TEM and the result is displayed in Figure 4. The normal endometrium in sham group was composed of monolayer columnar epithelial cells with microvilli on apical side. By contrast, the intact columnar epithelial cell of endometrium in the IUA group, with little microvilli on apical side, was wrinkled with a condensed chromatin, exhibiting a high-electron density in the nuclear zone, and some of nucleus has been cracked into several fragments (marked by long arrow). Besides, the rough-surfaced endoplasmic reticulums of these columnar epithelial cells expanded seriously, and some mitochondria became irregularly swollen, displaying the vacuole with fuzzy, and fractured edges. These changes of cells morphology indicated a status of cell denaturalization and inhibition of proliferation in IUA. However, after treatment with the free KGF solution or KGF-HP hydrogel group, these cells were recovered to a normal morphology with a clear cell boundary, normal mitochondria, and clear endoplasmic reticulums. Moreover, there were plenty of microvilli on the apical side of the EEC for these both groups, free KGF solution and KGF-HP hydrogel, but KGF-HP groups exhibited a more dense microvilli than free KGF or E2 group, indicating its more effective efficacy on IUA.

### 3.6 Receptive fertility recovery

**Figure 5.**
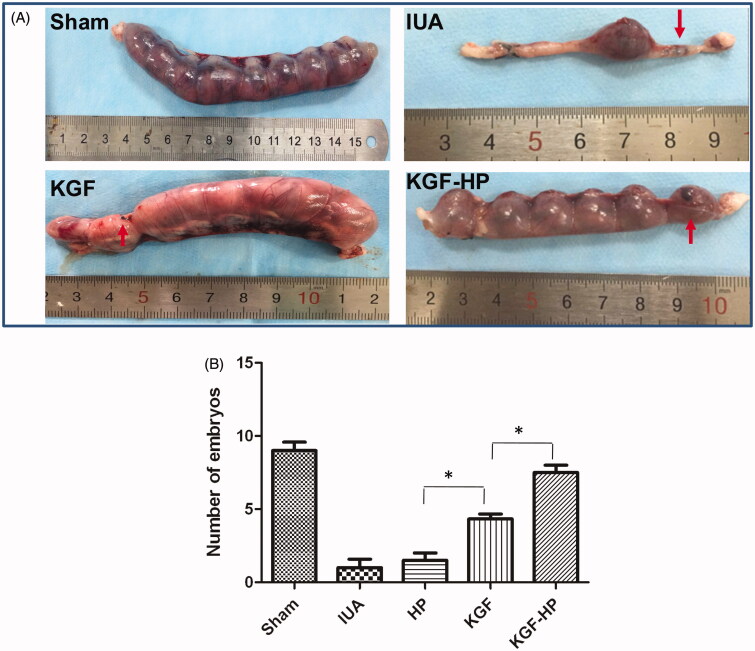
(A) Representative images of embryos implantation for rats treated by different formulations (the injured zone marked by red arrow), and (B) Pregnancies of different groups at 90 days postoperative in sham operated group, IUA group, KGF group, KGF-HP group. (*p < .05; *n* = 6).

The function recovery of the repair endometrium was assessed by testing whether it was receptive to a fertilized ovum and was able to support embryo development to the late stages of pregnancy. Ninety days after administration with KGF formulations, implanted embryos were found in some of the regenerated uterus, which were maintained to late a viable stage of pregnancy. Pregnancy rates were 66.6% in KGF-HP hydrogel group, 55.5% in KGF solution and 11.1% in IUA group compared to 100% in sham group (Figure 5). In addition, in KGF-HP hydrogel group or free KGF solution embryos were observed both in injured sites and normal sites, while no embryos were found in injured sites in IUA group. These results showed that KGF-HP hydrogel perfused into injured endometrium *in vivo* could reestablish functional maternal uterus to support the implantation of embryos and development of fetuses.

### 3.7 Regeneration of epithelium and angiogenesis of vascular

Repair and regeneration of columnar epithelial cells was very important for recovery of injured uterus. Therefore, Staining of Ki67 was firstly performed to evaluate the re-epithelialization of the injured endometrium. As shown in Figure S4(A,C), expression of Ki67 in IUA operation group was increased compared with the sham group, since cell proliferation as a self-protective effect is activated after tissue damage. There was also overexpression of Ki67 in both endometrial glandular epithelial cells and luminal epithelial cells at 7 d after treatment of KGF solution or KGF-HP hydrogel. However, level of total Ki67 expression in both types of cells was significantly higher than that in IUA group, even E_2_ group. Expectedly, the cell proliferation of uterus treated with KGF-HP hydrogel was more obvious than that of free KGF. These results were consistent with results of histological and functional evaluation.

Neovascularization in endometrium was helpful for regeneration of epithelium, considered as an important role for the restoration of functional fertility. The neovascularization at endometrium were characterized by immunohistochemical and immunofluorescence staining of CD31, a crucial vascular endothelial cells marker. Immunohistochemical staining is shown in Figure S4(B,D). There was little or no expression of CD-31 around injured zone of uterus in the IUA group or HP group. However, at 7 d after treatment with KGF-HP hydrogels, a large number of blood vessels but not microvessels were observed near the site of injury and number of blood vessel per field was significantly higher than other groups. Moreover, neovascularization distributed normally among functional layers in the endometrium of rats. Immunofluorescence of CD31 is displayed in Figure S5, the similar results, that was, KGF-HP hydrogel group exhibited the best angiogenesis of vessel. These results showed that KGF-HP could boost angiogenesis and lead to a rapid healing of injured uterus.

### 3.8 Inhibition of PI3KAKT-MTOR signaling pathway

**Figure 6.**
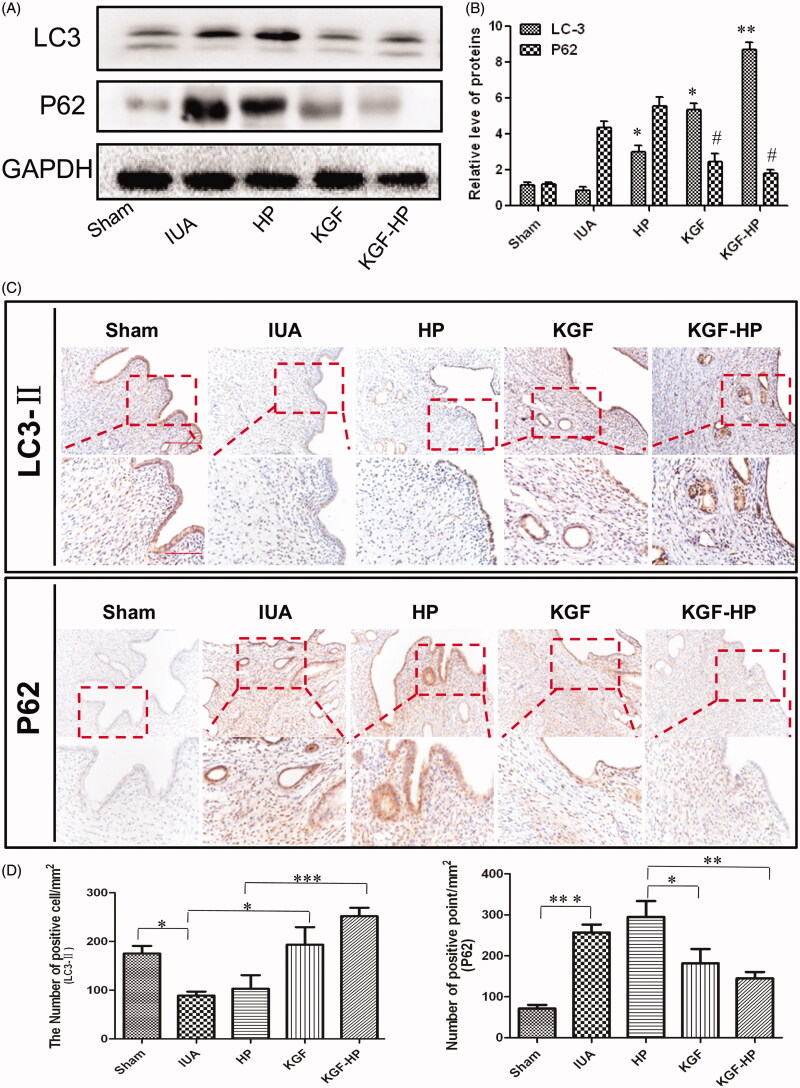
(A) Protein expressions of LC-3 and P62 in each group, (B) Quantification of western blot data, (C) Immunohistochemistry staining of LC3-II and P62 in each group, and (D) Quantification data of number of positive point of LC3-IIand P62 in each group. Original magnification: 200×; close-up magnification: 400×. (*p < .05; **p < .01; ***p < .001, *n* = 3).

To determine whether KGF and its receptor involved in the regulation of autophagy-associated proliferations of epithelial cell or angiogenesis of vascular, the typical proteins, such as LC3-II and P62 along PI3KAKT-MTOR signaling pathway were detected by Western blot. As shown in Figure 6(A), expression of LC3-II, an indicators of autophagy, were significantly increased after 7 d of treatment with KGF-HP hydrogel compared with other groups. Simultaneously, expression of P62, an important indicator of autophagic flux-related inhibition, in KGF-HP hydrogel group was drastically decreased in comparison with other groups (Figure 6(B)). Further, the immunohistochemical staining of both indicators were also performed and results are shown in Figure 6(C,D). The similar results were observed between these test groups. It was seen that expression-LC3-II was not only distributed on columnar epithelial cells but also localized on neovascular vessels for KGF-HP hydrogel. In contrast, there was not significant expression of P62 in injured endometrium after treatment KGF-HP hydrogel. These results showed a close relatively relationship between autophagy and proliferation of EEC and angiogenesis. KGF may play an important role in activation of the autophagic process, and thus promoted EEC and angiogenesis after IUA. Autophagy-associated repair of endometrial damage was also exhibited in previous publication (Sobolewska et al., 2009).

## 4. Discussion

In the years of women with reproductive capacity, normal endometrium undergoes about 400 cycles of self-repairing, including: shedding, regeneration, and differentiation and without scarring (Gargett, 2007). A general consensus has been reached that trauma and infection are the most common causes of IUA (Chomiski et al., 2016). At present, although clinical treatment using IUDs for IUA can restore the shape of the uterus cavity, it prevents normal endometrial regeneration and may increase risk of infection. Therefore, this therapeutic strategy results in a low rate of pregnancy, particularly in moderate and severe IUA. Currently, drug delivery systems especially *in situ* hydrogel can not only provide a physical barrier to prevent adhesion of injured uterus but also deliver the therapeutic agent in sustained release profile to repair injured tissues. It became a very popular treatment protocol of IUA (Liu et al., 2007; Cruz Orozco et al., 2012).

KGF is first well known for its role in wound healing and hair regeneration. It was firstly reported that KGF showed an important role in inducing keratinocytes migration and differentiation (7–10). Although various growth factors and cytokines including IL-6, TGF-β, GM-CSF, VEGF, PDGF, EGF, and FGF2 were secreted by dermal keratinocytes during self-healing of wound (Sugawara et al., 1997; Zhang, 2010 ), KGF was only produced by mesenchymal cells. In the meantime, KGF as a potent epithelial mitogen, the effects of cytoprotective and regenerative on epithelial tissues was deeply recognized and had been used in clinical applications. KGF was expressed specifically on epithelial cells after oxidant-induced lung injury and even at 11 d after injury it was also diffusely expressed in epithelium, indicating the protective or mitogen effect of KGF on the lung epithelium (Ray et al., 2003). KGF can promote survival and DNA repair of injured cells through binding to its high-affinity receptor, as a variant of FGF receptor 2 (FGFR2-IIIb) (Yang et al., 2010). FGFR2b is expressed by various types of epithelial cells of normal tissues including skin (Liu et al., 2016), intestine (Tarnawski & Ahluwalia, 2012), ovarium (Faustino et al., 2011), and endometrium (Ishikawa et al., 2008). After acute injury, expression of KGF is significantly up-regulated in various tissues including skin as well as the bladder (Slayden et al., 2000). It may indicate that KGF is most likely to be important for the healing of injured epithelia. Additionally, KGF was suggested to be a key regulator in organogenesis and angiogenesis (Gillis et al., 1999; Barbara et al., 2008). Endometrial KGF levels were increased by progesterone treatment in the macaque and mouse and are elevated during the luteal phase of the menstrual cycle in women. It was discovered that exogenous KGF stimulate spiral artery growth and inhibit glandular apoptosis during luteal-follicular transition, but did not affect cell proliferation in the endometrium or block menstrual sloughing and bleeding in normal uterus. But it was not clear whether exogenous KGF can facilitate the morphologic and functional recovery of the injured uterus. To investigate its effect on injured uterus, KGF, a heparin-affinitive growth factor, are also confronting with tremendous challenges in practical application because of its short half-life, poor stability, and low permeability.

Hydrogels are comprised of hydrophilic polymers dissolved in water forming a polymeric mesh concealing drug molecules inside (Nie et al., 2011). Additional to swell-ability, hydrogels encompass physical properties like mechanical resistance, permeability, surface features, and biocompatibility that can be altered through structural transformations (Schmolka, 1972). It was reported that the in situ poloxamer 407 hydrogel was potential vehicles to control the release of solid lipid nanoparticles (SLNs) (Dorraj & Moghimi, 2015). Poloxamer-407 has good temperature sensitivity, but lack of sufficient affinity for growth factors. In addition, the mechanical strength of conventional hydrogels is not suitable at normal body temperature, which may cause endometrial injury to be adhesive again after administration. Recently, a study explored the effect of KGF on the persistent proliferation and cornification of the vaginal epithelium when neonatal mouse vaginal was exposed to KGF during the estrogen-independent period (Masui et al., 2004). The results showed that treatment of KGF alone at doses less than 500 ng/ml did not induce permanent vaginal changes but such changes did occur in vaginae treated with heparin plus as little as 10 ng/ml KGF. In this study, temperature-sensitive HP was used as a hydrogel-forming material to support injured uterus and delivery of KGF. HP hydrogel not only exhibited a unique property of sol-gel transition at body temperature but also possessed a suitable apparent viscosity. These properties were required for HP hydrogels to be administrated as liquid forms at low temperature and adhere to endometrium at body temperature. The sponge-like and porous structures of HP hydrogels were observed by SEM image. These were highly associated with the crosslinking between multiple poly(ethyleneoxide) chains of HP (Mori et al., 2010). The micromorphology of HP not only played an important role in supporting normal lumen structure of the injured endometrium, but also controlled KGF release through these porous network structures. The release of KGF from HP hydrogels was significantly prolonged. The cumulative release from HP hydrogels was not more than 40% even within 7 d. The similar release behavior of aFGF, bFGF, and NGF from HP hydrogel for spinal cord and wounding were also reported in previous works (Wu et al., 2016; Zhao et al., 2016). The sustained release manner could be attributed to the –COOH and –OH groups of HP and bind to SH group of KGF (Rubin et al., 2001). The retention of KGF-HP hydrogels in uterine cavity was explored by in vivo bioluminescence imaging. Even at 7 day after administration, there was still a strong residual fluorescence in the uterine cavity for KGF-HP hydrogels, but a faint fluorescent was observed in KGF solution group. The enhanced retention of KGF-HP hydrogel was attributed to the strong interaction between HP hydrogel and endometrial adluminal surface. With these advantages, HP may be a promising candidate for delivering KGF to uterus for the injury endometrium.

In previous study, KGF can promote the growth of epithelial cells under physiological and pathological conditions (Li & Rinehart, 1998). Under the pathological condition, the growth promoting effect is beneficial to the tissue repair. In this study, the effects of exogenous KGF on the regeneration and repair of injured endometrium were carefully investigated *in vitro* and *vivo*. First, it was able to promote proliferation of H_2_O_2_-induced endometrial epithelial cells, which was confirmed by the cck-8 experiment. Interestingly, it was founded that the proliferation effect of KGF in EEC was not dose-dependent, exhibiting at optimal dose of KGF, 80 ng/ml. The proliferation effects of KGF on simian virus 40 (SV40)-immortalized human EEC was also not dose-dependent, with the optimum dose of 100 ng/ml (Li & Rinehart, 1998), which was similar to our study. Further, *in vitro* cell uptake experiment indicated that the use of HP hydrogels significantly enhanced the availability of KGF to EECs, resulting in more effective cells proliferation. The amount of KGF taken in by EECs in the presence of HP hydrogels was 3-folds higher than that of free KGF solution. There were similar report in these publication (Xu et al., 2016).With regard to the capacity for differentiation, survival, and motility, KGF has been of particular interest to repair the damaged endometrium by directly local delivery system (Eshghi et al., 2013).

Endometrial fibrosis is the primary pathological feature of IUAs characterized by excessive deposition and reorganization of extracellular matrix (ECM) replacing the normal endometrium (Liu et al., 2013). In this study, we successfully established a physiological and clinically relevant IUA rat model by mechanical injury by evaluating glandular loss and levels of fibrosis. HP hydrogel was very helpful to enhance retention and distribution of KGF in injured lumen of uterus compared with KGF solution. Afterward, the therapeutic effect of KGF on IUA was investigated in IUA rat model using E_2_ as positive control. Estrogen (E_2_), as an extensively studied for IUA treatment, is well known for postoperative hormone therapy to preventing recurrent adhesions (Cai et al., 2016). Estrogen stimulates proliferation of epithelial cells through interacting with the estrogen receptor α in cells. After treatment with KGF or KGF-HP hydrogels, we preliminary confirmed that KGF could improve morphologic recovery in the uterus. Moreover, it found that not only the number of glands was significantly increased but also the area of adhesions and tissue fibrosis was significantly mitigated after treatment with KGF-HP hydrogel. Especially to deserve to be mentioned, repair of glands in these groups was significantly better than that in E2 group at same dose level. Ultrastructure evaluations of injured uterus indicated that the injured EEC were recovered to a normal morphology with a clear cell boundary, normal mitochondria, and clear endoplasmic reticulums after KGF treatment. Moreover, KGF-HP groups exhibited more dense microvilli than free KGF or E2 group, indicating its more effective efficacy on IUA. Besides, KGF-HP hydrogel perfused into injured endometrium *in vivo* could reestablish functional maternal uterus to support the implantation of embryos and development of fetuses.

The roles of KGF in promoting morphologic and functional recovery of the injured uterus were evaluated from two aspects, cell proliferation and angiogenesis. Both endometrial glandular epithelial cells and luminal epithelial cells proliferated significantly at 7 d after treatment of KGF solution or KGF-HP hydrogel. Promotion of cells proliferation may be associated with a restorative microenvironment by decreasing inflammatory and immune responses (Gargett et al., 2008; Zhang et al., 2016), because it was reported that KGF decreased expression of TGF-β, a abundantly expressed inflammatory factor in the endometrium after injury of uterus (Carluccio et al., 2016). Angiogenesis, the growth of new capillaries from preexisting blood vessels, is essential to support the nutritional demands of tissues that are expanding or being repaired (Gillis et al., 1999). Because the uterus wall was rich in capillary networks, angiogenesis was important for the repair and regeneration of injured uterus. As many members of the FGF family have the function of promoting blood vessel growth (Wu et al., 2016). KGF as a direct stimulus of small blood vessels, stimulated the growth of microvascular endothelial cells, and induced the proliferation and migration of vascular endothelial cells in the literature.

A few studies have suggested that the autophagy may have a direct role in promoting cell proliferation in endometrium injury diseases (Choi et al., 2014). It was reported that KGF induced cell survival pathways by the activation of the multifunctional pro-survival Akt signaling axis *in vitro* and *in vivo* (Masui et al., 2004). In addition, the activation of autophagy by KGF was required to trigger early differentiation of keratinocytes in the publication (Belleudi et al., 2014). PI3KAKT-MTOR signaling pathway was highly associated with autophagy-induced cell proliferation. Several growth factors involved in cell survival was able to active/inhibit autophagy (Lin et al., 2011). Here, we investigated the autophagic role of KGF in proliferation of endometrial epithelial cells. LC-3-II was derived from the conversion of the cytosolic form of LC3-I, and represents a well-established marker for phagophores and autophagosomes. In this study, LC3-II protein levels were increased in KGF and KGF-HP group compared with IUA group. The results showed that the addition of KGF induced a significant increase of induce the autophagic response. To more carefully check the autophagic flux in KGF treatment group, the levels of the well-known autophagy substrate P62 was analyzed. Corresponding with the increasing expression of LC3- II, P62 was significantly decreased at day 7. Taken together, these changes suggest that a close relatively relationship between autophagy and proliferation of EEC and angiogenesis was firstly confirmed in injured uterus after KGF treatment. KGF takes part in activation of the autophagic process, and thus promoted proliferation of EEC and angiogenesis after IUA.

## Conclusions

In this study, a novel hydrogel with affinity to KGF was explored to prevent IUA, and promote morphologic and functional recovery of the injured uterus. The therapeutic effect on injured uterus was first demonstrated by comparing with E_2_. KGF was proved to play an important role in improving the proliferation of EEC and angiogenesis after uterus injury. Moreover, treatment with KGF-HP hydrogel produced a better effect on morphology and function recovery of the injured uterus due to the controlled release of KGF, comparing with treatment of free KGF solution. The underling mechanism was highly associated with activation of autophagy. Overall, KGF could be used as a new candidate for the treatment of IUA when combined with *in situ* hydrogel.

## Supplementary Material

IDRD_Helin_Supplemental_Content.doc
