# Supplementary material for: Temperature-sensitive heparin-modified poloxamer hydrogel with affinity to KGF facilitate the morphologic and functional recovery of the injured rat uterus
Source: Drug Deliv. 2017 Jun 2;24(1):867–81. doi: 10.1080/10717544.2017.1333173 (PMC8241134; doi:10.1080/10717544.2017.1333173)
Supplement: IDRD_Helin_Supplemental_Content.doc [file IDRD_A_1333173_SM0570.doc]

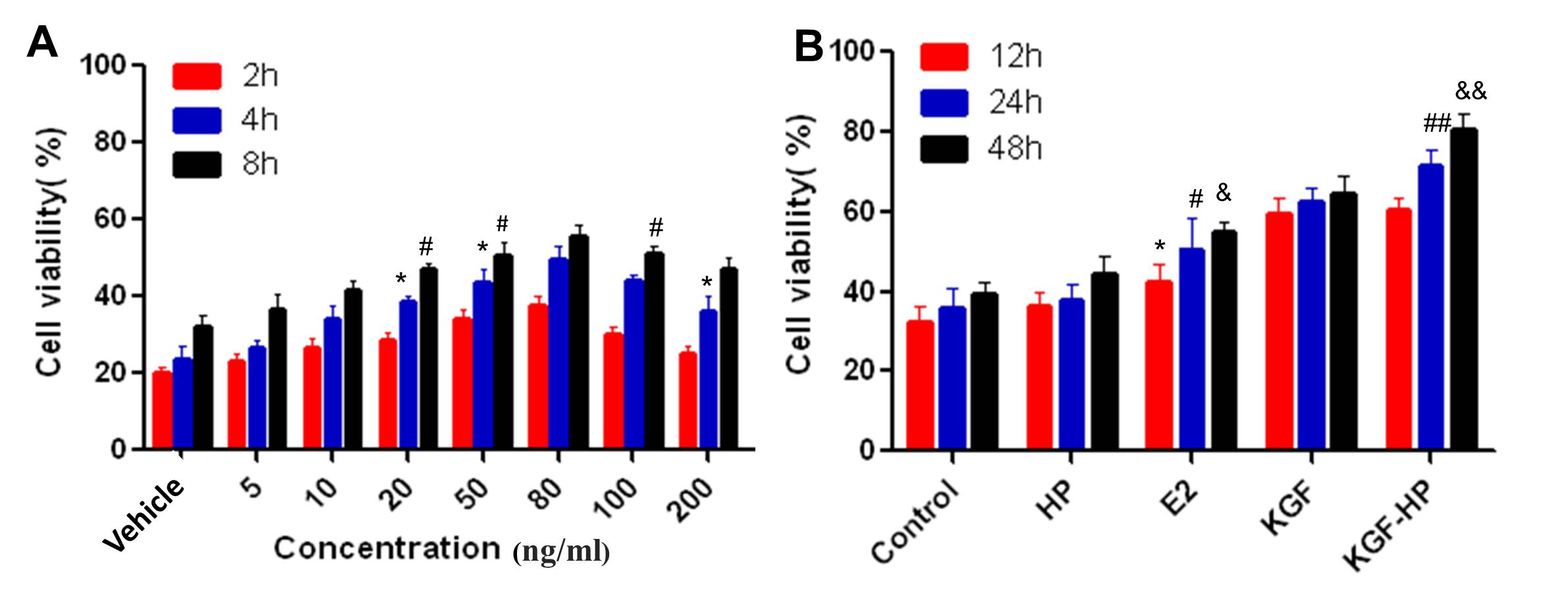


**Fig S1.** (A) Viability of the injured EEC after treated with different concentration of free KGF solution. (*: P<0.05 vs 80ng/ml, #: P<0.05vs 80ng/ml). (B) Cell proliferation effect of various treatments on EEC. (*: P<0.01vs KGF, #: P<0.05vs KGF, ##: P< 0.01vs KGF, &: P< 0.05vs KGF, &&: P< 0.01vs KGF).


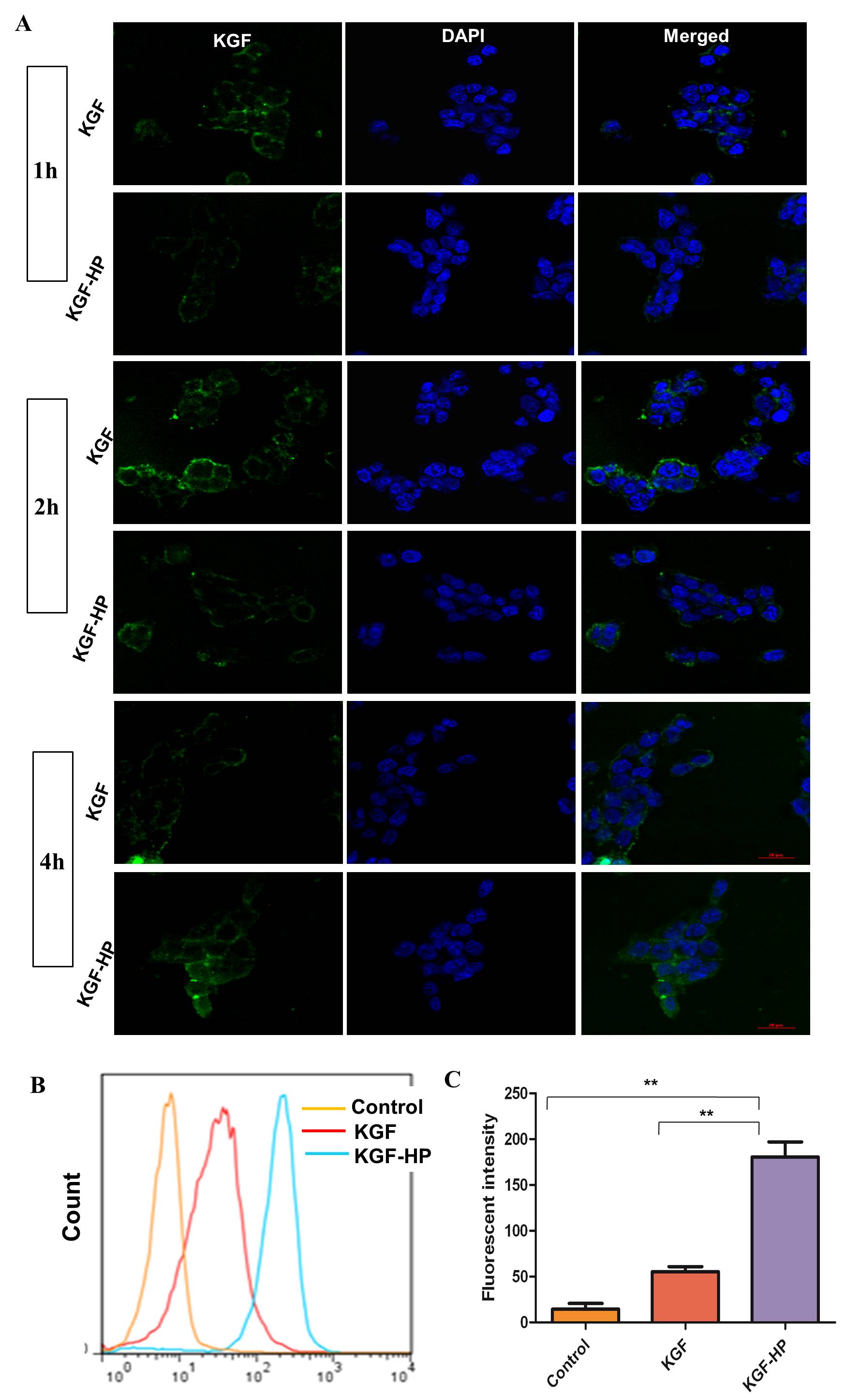


**Fig S2.** (A)CLSM images of EEC incubated with free KGF and KGF-HP hydrogel at a KGF concentration of 80ng/ml for 1h, 2h and 4h. (B) EEC uptake of KGF and KGF-HP by flow cytometry for 4h, respectively, (C) Quantitative analysis of intracellular uptake by flow cytometry. Scar= 20µm. (**:P<0.01)


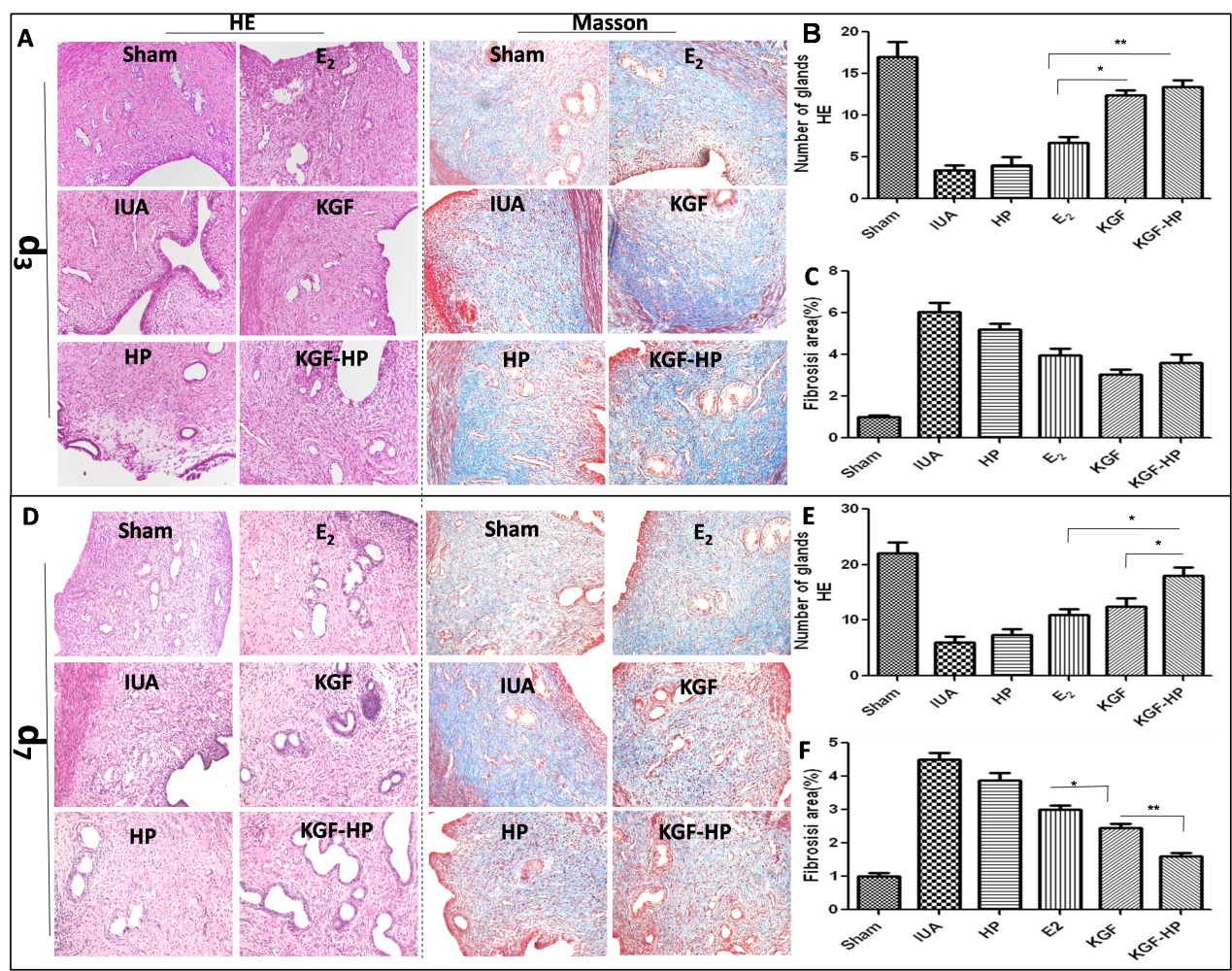


**Fig S3.** HE and Masson trichromestaining of the injuredendometriumaftertreatment with different therapies on day 3(A) and day 7 (D).In HE staining,Nuclei (blue), extracellular matrix andcytoplasm(red); In Masson’s trichrome, collagen(blue), cytoplasm and muscle (Red), the nucleus (violet). Original magnification: 200×.Quantification analysis of the glands number for each group at day 3 (B) and day 7(E); quantitative analysis the fibrosis area of each group at day 3(C) and day 7(F).

**Fig S4.** (A) Immunohistochemistry images of Ki67 (A), and CD31 (B) staining for injured uterus on day 7 after treatments. Rectangles refer to the close-up areas. Staining-positive cells were marked by red arrows (Scale bar 100 μm; magnification scale bar 50 μm). (C) Quantitative analysis of number of Ki67-positive cells (C), and (D) number of vessels per field at day 7 after surgery. Original magnification: 200×; close-up magnification: 400×. (*: P<0.05; **: P< 0.01; n=3)

**Fig S5.** Immunofluorescence staining **o**f CD31 (green) and DAPI (blue) in injured endometrium at day 7 after the different treatments. Scale bar =100μm.
